# Complementary evaluation of structure stability of perovskite oxides using bond-valence and density-functional-theory calculations

**DOI:** 10.1080/14686996.2018.1430449

**Published:** 2018-02-19

**Authors:** Ikuya Yamada, Akihiko Takamatsu, Hidekazu Ikeno

**Affiliations:** ^a^ Department of Materials Science, Graduate School of Engineering, Osaka Prefecture University, Sakai, Japan; ^b^ NanoSquare Research Institute, Research Center for the 21st Century, Organization for Research Promotion, Osaka Prefecture University, Sakai, Japan

**Keywords:** Bond valence sum, global instability index, DFT calculation, 60 New topics/Others, 107 Glass and ceramic materials, 401 1st principle calculations

## Abstract

Estimation of structure stability is an essential issue in materials design and synthesis. Global instability index (*GII*) based on bond-valence method is applied as a simple indication, while density functional theory calculation is adopted for accurate evaluation of formation energy. We compare the *GII* and total energy of typical *AB*O_3_-type perovskite oxides and rationalize their relationship, proposing that the criteria for empirically unstable structures (*GII* > 0.2 valence unit) correspond to the difference in total energy of 50–200 meV per formula unit.

## Graphical abstract

**Figure:**
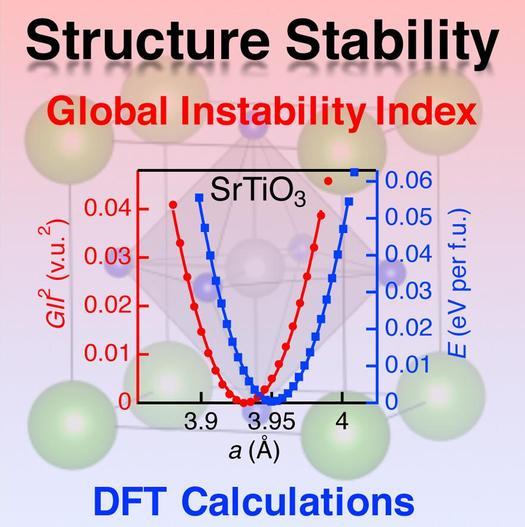

## 1. Introduction

Structure stability is an essential factor for evaluating and predicting materials including hypothetical compounds. For ionic compounds, ionic radii [1] and the radius ratios of cation to anion are primary indices to categorize and predict stable coordination polyhedra in crystal. Goldschmidt tolerance factor *t* [2] is extensively adopted to evaluate structure stability of perovskite structure: *t* = $\frac{r_{A}\;+\;r_{X}}{\sqrt{2}\left(r_{B}\;+\;r_{X}\right)}$, where *r*_*A*_, *r*_*B*_, and *r*_*X*_ are ionic radii of *A*, *B*, and *X* ions in *ABX*_3_ perovskite, respectively. When *t* is close to the unity, cubic perovskite (space group *Pm* $\overset{¯}{3}$ *m*) is stable at ambient conditions. When *t* is larger or smaller than the unity, distorted perovskite structures, typically GdFeO_3_-type orthorhombic perovskite, or phases other than perovskite (e.g. ilmenite) are expected instead of undistorted cubic perovskite. This index also serves to interpret systematic trends in structures and various properties for *AB*O_3_ oxides [3–6].

Bond valence method is widely applied to verify adequacy of structure refinement and also to estimate oxidation states of constituent ions [7,8]. Bond valences (*s*_*ij*_) are calculated by a formula: $s_{\mathrm{ij}}=\exp\left(\frac{l_{0}-l_{\mathrm{ij}}}{b}\right)$. *l*_*ij*_ is a bond length, *l*_0_ is a bond valence parameter empirically determined using experimental room-temperature structure data, and *b* is a parameter concerning bond softness [9]. The bond valence sum (*BVS*) is calculated as the sum of bond valences around the relevant ion: *BVS* = $\sum_{i=1}^{n}s_{\mathrm{ij}}$. *BVS* is approximately consistent with oxidation number of ion, thus enabling estimation of valences of constituent elements and cation/anion orderings in complex metal oxides [10–12]. *BVS*-based indices are utilized for evaluation of structural distortions; the bond discrepancy factor (*d*) is defined as the deviation of *BVS* from formal valence: *d*_*M*_ = *BVS*(*M*) – *V*_*M*_, where *V*_*M*_ is the formal valence of *M* ion. The *d*_*M*_ serves to estimate bond strains. When *d*_*M*_ > 0, the bonds around *M* ion are under compression stress (overbonding), whereas *d*_*M*_ < 0, under tensile stress (underbonding). The global instability index (*GII*) [13] is defined as root mean square of bond discrepancy index in the unit cell: $GII=\sqrt{\frac{\sum_{i=1}^{N}{\left(d_{i}\right)}^{2}}{N}}$, where *N* is the number of ions. *GII* is adopted for comparing structure instability in various perovskite forms including hypothetical phases [14,15]; *GII* = 0 means perfectly stable structures without steric distortions although structures with *GII* > 0.2 valence unit (v.u.) are uncommon [8]. The *GII*-based structure prediction program, SPuDS, developed by Lufaso et al. can calculate the degree and order of structure instability for various perovskite structures [15,16], as well as the tolerance factor. The close relationship between *GII* and tolerance factor was demonstrated by Zhang et al. [17]. The indices *d* and *GII* are also utilized for interpretation of structure-property relationship. Quadruple perovskite oxides *R*Cu_3_Fe_4_O_12_ (*R*: rare-earth metals) undergo two distinct electronic phase transitions, charge disproportionation (8Fe^3.75+^ → 5Fe^3+^  + 3Fe^5+^) and charge transfer (3Cu^2+^  + 4Fe^3.75+^ → 3Cu^3+^  + 4Fe^3+^) at low temperatures [18,19], the type of which is predominantly determined by strains on *R*–O and Fe–O bonds [20].

The above-mentioned indices derived from crystal structures are helpful because of their concise procedures, but precise evaluation of structure stability needs first-principle calculations. The most stable structure at the ground can be estimated by comparing total energies of polymorphs and even structural phase transition pressures are also available by using common tangent in total energy versus volume curves [21,22]. The stability of a crystal structure is evaluated by performing phonon calculations with harmonic approximation [23]. The presence of imaginary phonon modes tells the structure is dynamically instable, and their eigenvectors in the reciprocal space gives us possible transition pathway [24]. However, these procedures are rather complicated and require enormous computational time.

To our knowledge, most of structure stability evaluation studies are performed using either *BVS*-based indices or first-principle calculations. Complementary evaluation and integration of multiple indices improve structure prediction and resulting materials design. The aim of this paper is to evaluate structural stability of typical perovskite oxides by comparing *GII* and total energy. The empirical criteria (*GII* < 0.2 v.u. in stable structure) correspond to energy scale of < 50–200 meV per formula unit (f.u.) for perovskite structure using first-principles calculations.

## 2. Computational procedure

Since consideration of magnetic structures and spin states in density functional theory (DFT) calculation may prevent simple evaluation of structure stability, we selected typical nonmagnetic oxides for calculations as follows: CaTiO_3_, SrTiO_3_, BaTiO_3_, CaZrO_3_, SrZrO_3_, BaZrO_3_, NaTaO_3_, and LaAlO_3_. *BVS*s were calculated by using the following parameters: *l*_0_ (Å) = 1.651 for Al^3+^, 2.29 for Ba^2+^, 1.967 for Ca^2+^, 2.172 for La^3+^, 1.80 for Na^+^, 2.118 for Sr^2+^, 1.920 for Ta^5+^, 1.815 for Ti^4+^, 1.937 for Zr^4+^, and *b* = 0.37 Å for all [9]. Structure optimizations using *GII* were performed; the *BVS*s were calculated by changing lattice constant *a* by 0.05 Å step around the optimized unit cell size (*a* = *a*_0_) with minimum *GII* value.

Single-point energy calculations for SrTiO_3_ and BaZrO_3_ in cubic perovskite structure were performed using cubic unit cell of length *sa*_exp_ with fixed internal coordinates, where *a*_exp_ is the experimental lattice constant, and *s* is the scaling factor changed by 0.001 step around unity. The calculations were performed using the plane-wave-based augmented projector wave (PAW) method as implemented in the VASP code [25–29]. The exchange–correlation interaction was treated as a generalized gradient approximation (GGA) using the function formulated by Perdew, Burke, and Ernzerhof [30]. The PAW potential data-set with radial cutoffs of 1.45, 2.3, 2.8, 2.5, 2.8, 1.9, 2.8, 2.5, 2.9 and 1.52 Bohr for Na, Ca, Ba, Sr, La, Al, Ti, Zr, Ta and O were employed, respectively, where Na-3s, 3p, Ca-3s, 3p, 4s, Ba-5s, 5p, 6s, Sr-4s, 4p, 5s, La-5s, 5p, 6s, 5d, Al-3s, 3p, Ti-3d, 4s, Zr-4s, 4p, 5s, 4d, Ta-6s, 5d, and O-2s, 2p were described as valence electrons. The plane wave cut-off energy was set to 550 eV, and 12 × 12 × 12 *k*-points mesh with the Monkhorst-Pack scheme was used for integration in the reciprocal space. The minimum value was set to zero in the relative total energy.

## 3. Results and discussion

**Figure 1.**
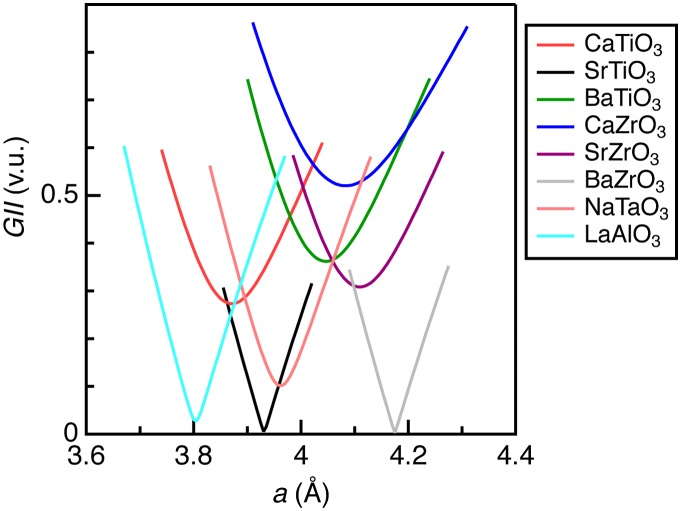
Global instability index for typical *AB*O_3_-type metal oxides in cubic perovskite structure.

Figure 1 shows calculated *GII* for the selected *AB*O_3_-type oxides. Only two oxides, SrTiO_3_ and BaZrO_3_, had the minimum *GII* values close to zero, whereas others did not reach near zero (< 0.01 v.u.). SrTiO_3_ (*t* = 1.001) and BaZrO_3_ (*t* = 1.004), which crystallize in cubic perovskite at ambient conditions, displayed linear behavior in *GII* versus *a*. LaAlO_3_ (*t* = 1.009) exhibited an almost linear curve down to the minimum value (*GII* = 0.027 v.u.), whereas it crystallizes in the rhombohedral perovskite structure at ambient conditions. This implies that the cubic perovskite phase is nearly stable for this oxide, as expected from the *t* value close to the unity. NaTaO_3_ (*t* = 0.967, orthorhombic perovskite at ambient conditions), which had a smaller *GII* value (0.102 v.u.) next to LaAlO_3_, demonstrated slightly nonlinear behavior. Other oxides with larger *GII* values (>0.1 v.u.) at the minima showed quadratic-like dependences and the deviations from a linear curve were apparently enhanced as the minimum *GII* increased. These arguments imply that the *GII* values hardly apply to these oxides because the energy is proportional to square of *GII* in principle [8].

**Figure 2.**
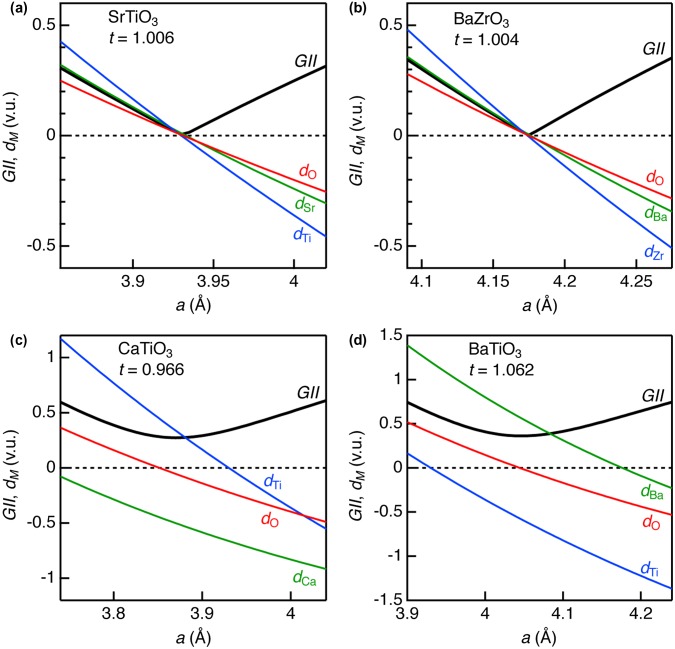
Global instability index and discrepancy factors for selected *AB*O_3_-type metal oxides in cubic perovskite structure; (a) SrTiO_3_, (b) BaZrO_3_, (c) CaTiO_3_, and (d) BaTiO_3_.

Figure 2 illustrates *GII* and *d*_*M*_ as a function of *a* for *A*TiO_3_ (*A* = Ca, Sr, and Ba) and BaZrO_3_. For any *A*, *B*, and O ions in all the oxides, bond discrepancy monotonically increased when *a* decreased. This corresponds to overbonding in *A*–O and *B*–O bonds in compression stress (or underbonding in tensile stress). *GII* converged to almost zero (0.006 v.u.) at *a*_0_ = 3.930 Å for SrTiO_3_, as well as near zero values for *d*_Sr_, *d*_Ti_, and *d*_O_. This result indicates that the optimized crystal structure could be obtained for this oxide, which is in agreement with the literature [31]. Also, BaZrO_3_ displayed similar behavior with SrTiO_3_; *GII* = 0.003 v.u. at *a*_0_ = 4.175 Å when the structure optimization was achieved. The *a*_0_ values obtained at *GII* minima were almost identical with the experimental ones for room-temperature cubic perovskite phases (Table 1).

**Table 1.** Tolerance factor (*t*), ionic radius of *A*- and *B*-ions, *GII* for cubic (*GII*_cubic_) and distorted perovskite structures (*GII*_exp_), *a*_0_, and crystal structure at room temperature determined by experiment for selected *AB*O_3_-type oxides.

Oxide	*t*	*r*_*A*_ (Å)	*r*_*B*_ (Å)	*GII*_cubic_ (v.u.)	*a*_0_ (Å)	*GII*_exp_ (v.u.)	Crystal system, space group, and lattice constants (Å) determined by experiment
CaTiO_3_	0.966	1.34	0.605	0.273	3.870	0.128	Orthorhombic (*Pbnm*); *a* = 5.3789, *b* = 5.4361, *c* = 7.6388 [32]
SrTiO_3_	1.001	1.44	0.605	0.006	3.930	0.102	Cubic (*Pm*$\overset{¯}{3}$*m*); *a* = 3.9049 [33]
BaTiO_3_	1.062	1.61	0.605	0.362	4.045	0.410	Tetragonal (*P*4*mm*); *a* = 3.9925, *c* = 4.0365 [34]
CaZrO_3_	0.914	1.34	0.72	0.521	4.085	0.140	Orthorhombic (*Pbnm*); *a* = 5.5890, *b* = 5.7586, *c* = 8.0140 [35]
SrZrO_3_	0.947	1.44	0.72	0.309	4.110	0.156	Orthorhombic (*Pbnm*); *a* = 5.7862, *b* = 5.8151, *c* = 8.1960 [36]
BaZrO_3_	1.004	1.61	0.72	0.003	4.175	0.049	Cubic (*Pm*$\overset{¯}{3}$*m*); *a* = 4.1879 [37]
NaTaO_3_	0.967	1.39	0.64	0.102	3.960	0.103	Orthorhombic (*Pbnm*); *a* = 5.48109, *b* = 5.52351, *c* = 7.79483 [38]
LaAlO_3_	1.009	1.36	0.535	0.027	3.805	0.078	Rhombohedral (*R*$\overset{¯}{3}$*c*); *a* = 5.48109, *c* = 7.79483 [39]

Since tolerance factors for these two oxides are almost the unity, the *GII* is simple index for stable structures. When *t* is smaller than the unity [e.g. *t* = 0.966 in CaTiO_3_, see Table 1 and Figure 2(c)], the structure optimization via *GII* could not be well conducted in cubic perovskite structure. The minimum *GII* = 0.273 v.u. was obtained at *a*_0_ = 3.870 Å for CaTiO_3_, which is much larger than the empirically determined threshold value (0.2 v.u.). The structural instability of this oxide is explained by underbonding of Ca–O bonds (*d*_Ca_ = −0.500 v.u.) and overbonding of Ti–O (*d*_Ti_ = 0.338 v.u.) in the *GII*-optimized structure, whereas O ions are in insignificant stress (*d*_O_ = −0.054 v.u.). As a result, structure transformations including rotations of the TiO_6_ octahedra occur, as evidenced by the fact that CaTiO_3_ crystallizes in GdFeO_3_-type orthorhombic perovskite structure at ambient conditions (room temperature and atmosphere pressure). BaTiO_3_, whose *t* is larger than the unity, also did not converge into stable cubic perovskite structure. Unlike CaTiO_3_, overbonding of Ba–O bonds (*d*_Ba_ = 0.570 v.u.) and underbonding of Ti–O bonds (*d*_Ti_ = −0.576 v.u.) were predominant in the *GII*-optimized structure. This oxide actually crystallizes in tetragonal perovskite structure at ambient conditions. The above results demonstrate that *GII* and *t* are consistent, confirming their usefulness as structure instability indices.

**Figure 3.**
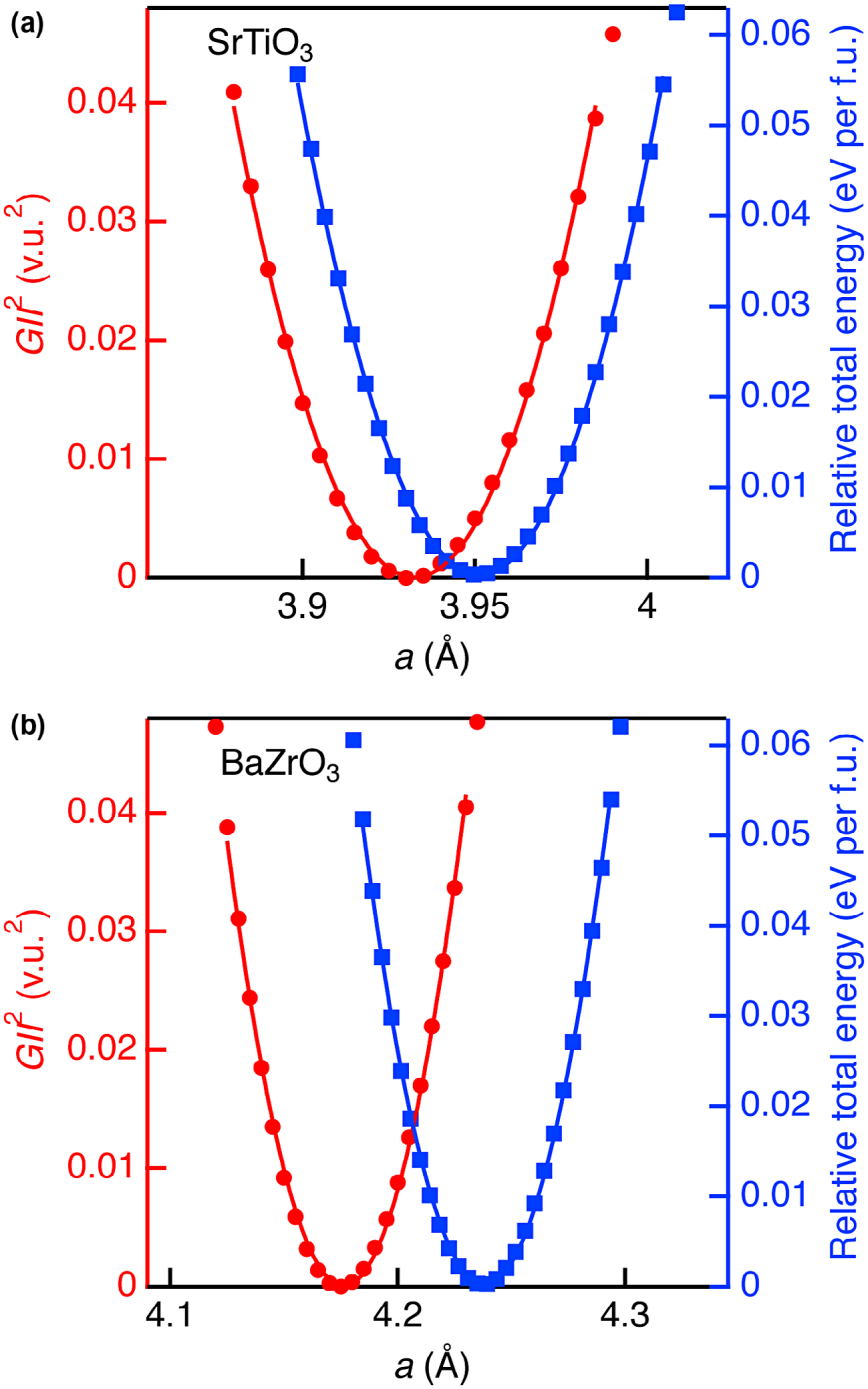
Square of global instability index (red circles) and relative total energy (blue squares) as a function of *a* for (a) SrTiO_3_ and (b) BaZrO_3_ in cubic perovskite structure. The curves represent the fitting results using quadratic function.

Figure 3 shows comparison between *GII* ^2^ and relative total energy (*E*_total_ in eV/f.u.) for SrTiO_3_ and BaZrO_3_. The optimized lattice constants of SrTiO_3_ and BaZrO_3_ by DFT calculations with minimum total energy were overestimated from experimental value by about 1.5%. This is mainly ascribed to the GGA used in the calculation to express the exchange-correlation energy. However, the change of total energy induced by the lattice strain can be calculated in better accuracy because significant part of the errors in exchange-correlation energy introduced by GGA is canceled out. In both oxides, *GII* ^2^ and *E*_total_ could be fitted by using the quadratic function fitting: *GII* ^2^ = *k*_1_(*a* – *a*_0_)^2^ + *G*_0_^2^ and *E*_total_ = *k*_2_(*a* – *a*_0_)^2^, where *a*_0_ is the unit cell edge when *GII* ^2^ or *E*_total_ is the minimum, and *G*_0_ is the minimum *GII* value. Fitting range were limited within the *GII* < 0.2 v.u. (*GII* ^2^ < 0.04 v.u.^2^). The obtained coefficients *k*_1_ and *k*_2_, and resulting *k*_2_/*k*_1_ are listed in Table 2. The *k*_1_ values for SrTiO_3_ and BaZrO_3_ are almost identical, and *k*_2_ also similar. The *k*_2_/*k*_1_ values are approximately 1.3 eV/(v.u.^2^ f.u.). Note that this is the first example of the conversion coefficient between *GII* and *E*_total_ in cubic perovskite structure, enabling total energy estimation as *E*_total_ = ~1.3 × *GII* ^2^ eV/f.u.

**Table 2.** Fitting results of *GII* ^2^ and relative total energy using quadratic function for SrTiO_3_ and BaZrO_3_.

	*k*_1_ [v.u.^2^/Å^2^]	*k*_2_ [eV/(Å^2^ f.u.)]	*k*_2_*/k*_1_ [eV/(v.u.^2^ f.u.)]
SrTiO_3_	14.41(14)	19.44(10)	1.349
BaZrO_3_	14.34(14)	17.69(8)	1.234

**Figure 4.**
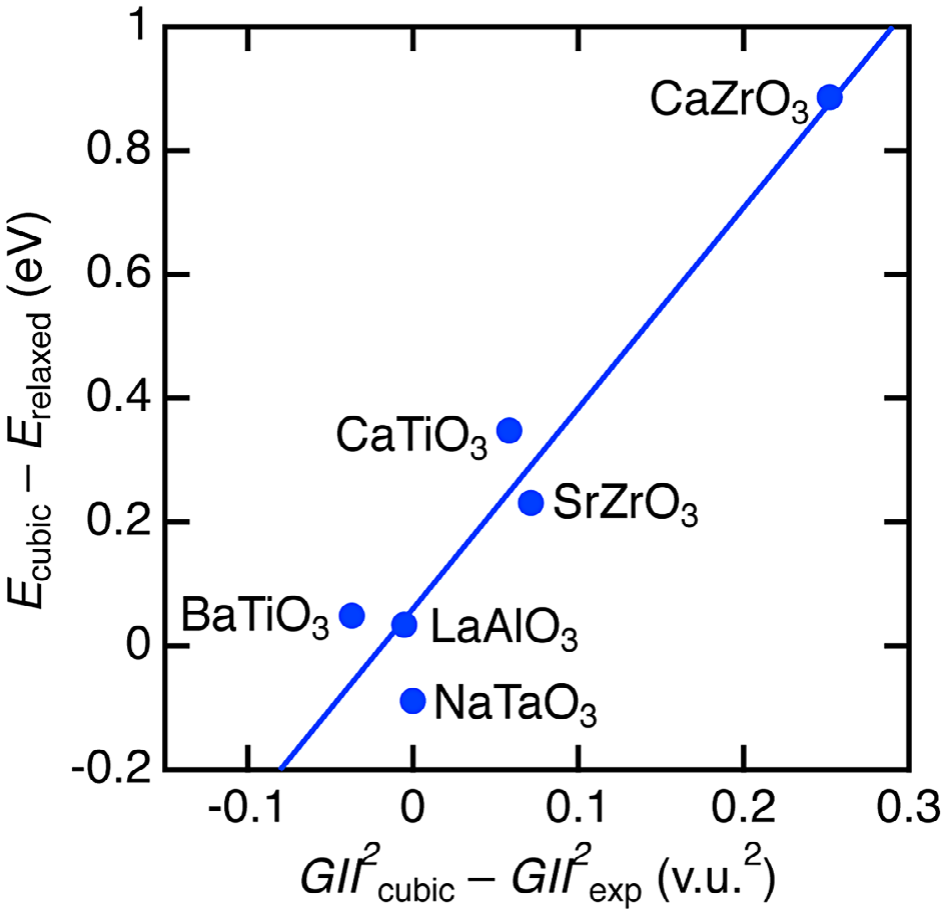
Comparison of structure instability between total energy and *GII*. The line represents the linear fitting result.

Furthermore, we tentatively evaluated the instability for severely unstable structures. The instability of hypothetical cubic perovskite structure in Figure 1 was estimated from the differences in *GII* ^2^ between the minimum values in cubic structure (*GII* ^2^_cubic_) and values for experimentally observed structures (*GII* ^2^_exp_) [32–39]. The instability in total energy was estimated by the difference between the energy at the minimum for cubic structure (*E*_cubic_) and the energy at relaxed non-cubic perovskite structure by DFT. Figure 4 demonstrates the correlation between the DFT- and *GII*-based instability. Rough estimation by linear fitting represents a relationship of *E*_total_ = 0.06(5) + 3.2(5) × *GII* ^2^ eV/f.u. The coefficient is about 2.5 times larger than those obtained by the analysis of stable cubic phases in Figure 3, but their scales are at the same level. Large-scale data analysis is needed to improve the total energy-*GII* relationship, but it is beyond the aim of the present study.

It should be noted that the *GII*s were calculated using bond-valence parameters, which were empirically determined by the structures at room temperature. On the other hand, total energies obtained calculated by DFT represent the internal energies at 0 K; the temperature dependence of internal energy and entropy term in the Helmholtz free energy are not taken into account. The changes of free energy introduced by lattice strain can be estimated quantitatively by performing phonon calculations under quasi-harmonic approximation if a target compound is dynamically stable [24,40]. Reflecting the thermal expansion of a crystal, the equilibrium volume at finite temperature slightly shifted from that at 0 K. The curvature of energy-volume curve (similar to *k*_2_ in this manuscript) also changes as increasing the temperature. However, the changes of these quantities at room temperature from those at 0 K are insignificant (order of 0.1%).

We verified adequacy of structure instability obtained from *GII* and *E*_total_ calculations. It is known that stable structures with *GII* > 0.2 v.u. are rare. This *GII* value corresponds to energy instability by ~50 meV/f.u according to the relationship obtained from cubic perovskites (SrTiO_3_ and BaZrO_3_) in Figure 3, whereas ~200 meV/f.u. was obtained from the analysis of non-cubic perovskites (CaTiO_3_, BaTiO_3_, CaZrO_3_, SrZrO_3_, NaTaO_3_, and LaAlO_3_) in Figure 4. To our knowledge, there are no established criteria to judge instability in total energy but energy differences among perovskite polymorphs are usually obtained by the order of several ten meV [41–44]. Therefore, *GII* calculations can reasonably exclude unstable phases at low cost.

## 4. Conclusions

We compared *GII* on bond-valence model and total energy by DFT calculations for cubic perovskite oxides. The obtained conversion coefficient is in roughly agreement with the energy scale in the previous DFT calculations, supporting the adequacy of *GII* as structure instability index. As well as ionic radii and Goldschmidt tolerance factor, *GII* can be adopted for structure-instability evaluation and prediction. For further study, integration of the above indices based on large-scale data analysis is expected to achieve fast and reliable materials design and exploration.

## Funding

This work was supported by the Grants-in-Aid for Scientific Research from the Japan Society for the Promotion of Science, the Ministry of Education, Culture, Sports, Science and Technology of Japan [grant numbers 26106518; 16H04220; 16H00893; 17K19182].

## Disclosure statement

No potential conflict of interest was reported by the authors.
